# On-Chip Planar Metasurfaces for Magnetic Sensors with Greatly Enhanced Sensitivity

**DOI:** 10.1021/acsnano.5c00422

**Published:** 2025-03-06

**Authors:** Aleix Barrera, Emile Fourneau, Natanael Bort-Soldevila, Jaume Cunill-Subiranas, Nuria Del-Valle, Nicolas Lejeune, Michal Staňo, Alevtina Smekhova, Narcis Mestres, Lluis Balcells, Carles Navau, Vojtěch Uhlíř, Simon J. Bending, Sergio Valencia, Alejandro V. Silhanek, Anna Palau

**Affiliations:** † Institut de Ciència de Materials de Barcelona, ICMAB-CSIC, Campus de la UAB, Bellaterra 08193, Spain; ‡ Experimental Physics of Nanostructured Materials, Q-MAT, Department of Physics, Université de Liège, Sart Tilman B-4000, Belgium; § Departament de Física, Universitat Autònoma de Barcelona, Bellaterra 08193, Spain; ∥ CEITEC BUT, Brno University of Technology, Purkyňova 123, Brno 61200, Czech Republic; ⊥ Helmholtz-Zentrum Berlin fur Materialien und Energie Albert-Einstein-Strasse 15, Berlin D-12489, Germany; # Institute of Physical Engineering, Brno University of Technology, Technická 2, Brno 616 69, Czech Republic; ¶ Centre for Nanoscience and Nanotechnology, Department of Physics, University of Bath, Bath BA2 7AY, U.K.

**Keywords:** magnetic flux concentrators, metamaterials, metasurfaces, micromagnetism, magnetic sensors

## Abstract

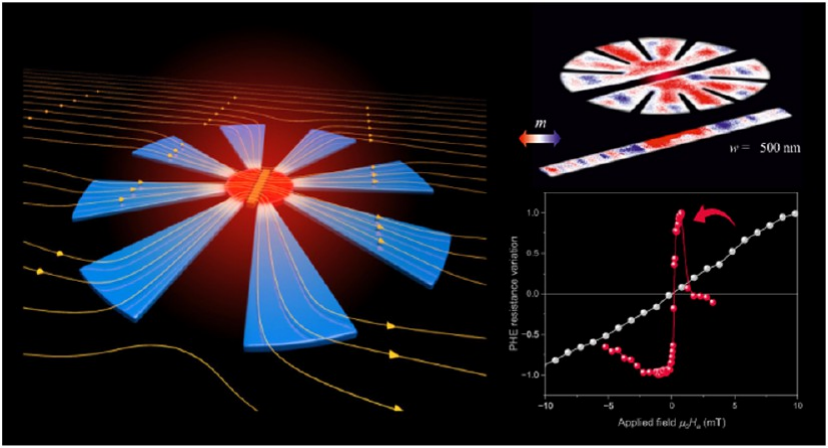

Metamaterials with engineered structures have been extensively investigated for their capability to manipulate optical, acoustic, or thermal waves. In particular, magnetic metamaterials with precise geometry, shape, size and arrangement of their elemental blocks may be used to concentrate, focus, or guide magnetic fields. In this work, we show the potential of using soft-magnetic permalloy (Py) metasurfaces to tailor the physical properties of other magnetic structures at the local scale. As an illustration, the magnetic response of a Cobalt (Co) sensor bar placed at the core of a Py metasurface is investigated as a function of in-plane magnetic fields through the planar Hall effect. Our findings reveal that by appropriately selecting the metasurface geometrical parameters, we can adjust the Co bar’s coercive field and susceptibility, leading to a huge enhancement in sensor sensitivity of over 2 orders of magnitude. Micromagnetic simulations, coupled with magneto-transport equations and X-ray photoemission electron measurements (XPEEM) with contrast from magnetic circular dichroism (XMCD), accurately capture this effect and provide insights into the underlying physical mechanisms. These findings can potentially enhance the performance and versatility of magnetic functional devices by using specifically designed structural magnetic materials.

## Introduction

Controlling magnetic spin textures such as domain walls, vortices, or skyrmions is crucial for advancing multiple emerging technologies.^1,2^ In a broader context, understanding and manipulating magnetic micro- and nanostructures represents a rich and continuously growing area of research due to their wide range of applications in data storage, information processing, magnetic field sensing, biomedicine, catalysis, etc.^3−6^ Key to the success of such devices is the careful design of their magnetic stability and operational efficiency. Achieving highly stable magnetic states typically requires the use of large magnetic fields or currents for operation. Alternatively, effective magnetic state manipulation can be achieved by using different physical phenomena such as electric fields,^7^ laser pulses,^8^ or strain^9^ as external stimuli, among many others. Moreover, extensive effort has been devoted to modulating the magnetic properties of low dimensional structures through material and shape engineering.^4,10^ In this way, important magnetic properties like susceptibility, coercivity or remanent magnetization can be adjusted to improve the performance or add new functionalities to emerging technologies that rely on magnetic devices. Magnetic sensors that utilize magnetoresistive effects have attracted significant attention due to their simple design, easy integration, and relatively high sensitivity compared to alternative methods.^11,12^ Ongoing efforts target improved sensitivity, focusing on increasing the magnetoresistance ratio or reducing the saturation field.^11,12^ One promising approach involves boosting the magnetic field at the sensing point by placing the sensor in the gap between two ferromagnetic pieces. The original idea of achieving flux concentration using soft magnetic materials shaped in specific ways was first introduced in the design of electromagnets, where the magnetic field in the gap could be enhanced by altering the shape of the yoke acting as a flux concentrator.^13^ While this concept has been widely explored to enhance the sensitivity of magnetic sensors,^14−16^ its full potential has not yet been realized. Therefore, it is highly desirable to explore innovative approaches that could control the response of magnetic structures at low-applied fields for the development of high-sensitivity, energy-efficient, and cost-effective magnetic sensors and functional devices.^12,17^

Metamaterials and metasurfaces, engineered materials structured at subwavelength scale, have attracted increasing attention due to their unprecedented capability to manipulate electromagnetic, optical or thermal waves,^18−20^ thus opening the door to new and unique functionalities.^21^ Most electromagnetic metamaterial designs are based on resonant elements intended to operate at microwave frequencies and above. However, the concept of metamaterials, particularly those designed using magnetostatic transformation optics, has significantly broadened the possibilities for manipulating low-frequency electromagnetic waves and static fields.^22,23^ The solutions for low-frequency electromagnetic waves present unique advantages compared to other regions of the electromagnetic spectrum. First, in the static limit, magnetic and electric fields decouple. This allows for comprehensive control of magnetic fields by using nonresonant materials with tunable values of magnetic permeability.^24^ In this regime, it is possible to develop all-magnetic metamaterials that can manipulate the magnetic field’s direction and intensity. This is achieved via transformation optics involving a coordinate transformation, which preserves the form of Maxwell’s equations but affects the components of the permittivity and permeability tensors. The fundamental ingredient needed to achieve efficient guidance of magnetic field lines and negligible external distortion of a uniformly applied field is a highly anisotropic magnetic permeability tensor μ. More precisely, this condition can be fulfilled in an axially symmetric structure by combining a radial (μ_ρ_) and angular (μ_θ_) relative permeability components fulfilling the relations μ_ρ_μ_θ_ = 1 and μ_ρ_ ≫ μ_θ_. Recognizing that natural materials that satisfy these conditions do not exist, scientists have proposed several metamaterials constructed from alternating layers or wedges to serve as approximations. These materials strategically combine superconducting wedges to suppress the azimuthal permeability and ferromagnetic wedges to enhance μ_ρ_ and thus tailor their effective permeabilities to enable the desired electromagnetic behavior.^25^ Second, since the static case effectively corresponds to an infinitely large wavelength, there are fewer constraints on the dimensions of the metamaterial components or the overall device. Anisotropic metamaterials consisting of an array of superconducting plates have been implemented as noninvasive shielding of weak magnetic fields.^22^ Later, hybrid systems consisting of different arrangements of ferromagnetic shells with alternated layers or wedges were shown to cloak,^26−29^ compress,^30,31^ guide or couple^32^ magnetic fields.

Magnetic metamaterials able to concentrate static or low-frequency magnetic fields have been modeled and experimentally tested using three-dimensional, long cylindrical shells made from concentric, radially aligned, equidistant alternating wedges of soft ferromagnetic material. In these systems, the magnetic field can be enhanced by a factor that depends on the ratio of the outer/inner shell radii and the number of funnels.^30^ Building on this design, recent advancements have shown the potential of planar metasurfaces for magnetic flux concentration. By combining the effects of metamaterial concentrators and the demagnetizing fields in finite geometries, a significant increase in magnetic field concentration can be achieved. These structures utilize flower-shaped metasurfaces that feature alternating ferromagnetic petals, analogous to the long wedges used in three-dimensional metamaterials. This innovation opens up new technological possibilities to improve the performance of small magnetic sensors and develop multifunctional magnetic devices^33−35^

In this research, we leverage the exceptional characteristics of metamaterials to manipulate the magnetic behavior of a small cobalt bar located at the center of a tailored magnetic flux concentrator (MFC) based on planar metasurfaces in a flower-shaped configuration. We show that it is feasible to control the reversal of the Co bar magnetization by adjusting various geometrical parameters such as shape, size, number of petals, and thickness of the metasurfaces. Furthermore, we have found that the sensitivity of bar-shaped sensors based on the magnetoresistance Planar Hall Effect (PHE) can be greatly improved by up to 2 orders of magnitude. Deeper insights into the mechanism governing the magnetic flux concentration in the metasurfaces are obtained by space-resolved imaging of the magnetic domain structure via X-ray photoemission microscopy (XPEEM). The experimental findings are in agreement with micromagnetic simulations that replicate both the XPEEM and PHE outcomes.

## Results and Discussion

**Figure 1:**
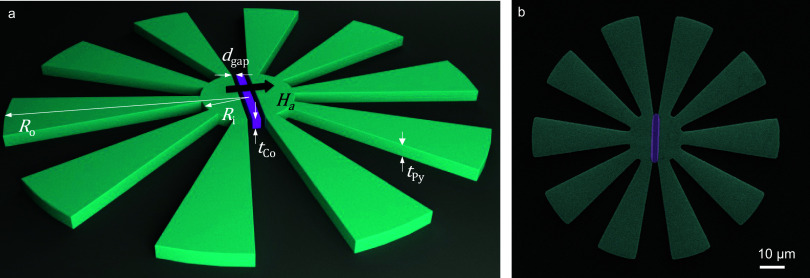
(a) Schematic illustration of a typical magnetic metasurface used for magnetic field concentration indicating the main geometrical parameters and the applied magnetic field orientation. (b) False-color SEM image of an actual device with 10 petals, zero gap, *R*_i_ = 10 μm, *R*_i_ = 10 μm, and *R*_0_ = 50 μm.

The magnetic metasurfaces under consideration comprise a flower-shaped structure of Permalloy (Py) with thickness *t*_Py_ deposited on a Si substrate, as depicted in the schematic representation of Figure 1(a). The fabrication process is detailed in the Methods Section. The structures feature a central core with a radius of *R*_i_, surrounded by an evenly spaced number of petals, *N*_p_, with an external radius *R*_0_. A ferromagnetic Cobalt (Co) bar with a thickness *t*_Co_, width *w*, and length *L* = 2*R*_i_, is situated in a rectangular slot patterned inside the core. The gap separating the bar from both sides of the MFC is defined as *d*_gap_. Designs with various dimensions (*R*_i_, *R*_0_, *t*_Py_, *N*_0_) and *d*_gap_ values were fabricated (see Table 1). All the measurements were performed with the magnetic field applied in the plane and perpendicular to the long eas*y*-axis (EA) of the Co bar. A scanning electron microscopy (SEM) image of a typical device is shown in Figure 1(b).

**Table 1:** Dimensions of Co Sensor Bars (Thickness, *t*_Co_, Width, *w*, and Length, *L* = 2 *R*_i_) and Py Metasurfaces (Inner Ratio, *R*_i_, Radii Ratio, *r* = *R*_o_/*R*_i_, Thickness, *t*_Py_, Gap *d*_gap_, and Number of Petals, *N*_p_) Used for Experimental Measurements

figure	*t*_Co_ (nm)	*w* (μm)	*R*_i_ (μm)	*r* = *R*_0_/*R*_i_	*t*_Py_ (nm)	*d*_gap_ (nm)	*N*_p_
Figure 2e,g	50	5	10	10	60	0	10
Figure 3d–f	60	5	25	5	100–300	30	10
Figure 4a,b	60	5	25	1.5–10	60–160	30	10
Figure 4c,d	60	5	25	5	100	30–6000	10
Figure 4e,f	60	5	25	2.5–10	115	30	2–40
Figure 6a,d	60	5	25	1.5–10	160	30	10
Figure 6b,e	60	5	25	5	100	30–6000	10
Figure 6c,f	60	5	25	5	115	30	2–40
Figure 7e	60	5	25	5	100	30	16
Figure 8d–g	60	0.3–5	1–25	3	100	30	10
Figure 9	50	0.4	0.5	3	60	0	10

### Magnetic Characterization

**Figure 2:**
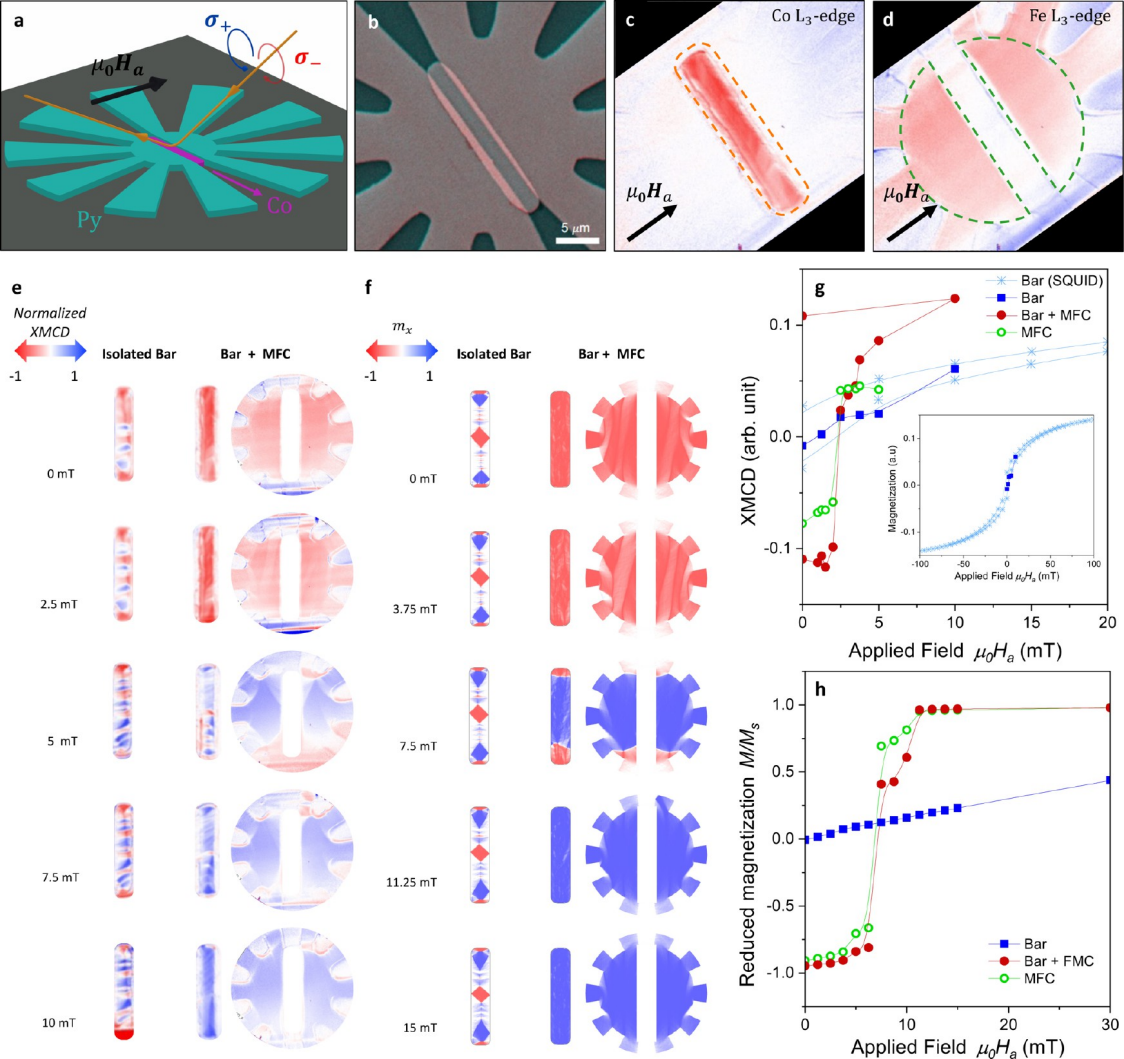
(a) Schematic representation of the XPEEM experiment employing X-ray Magnetic Circular Dichroism (XMCD) for magnetic contrast. (b) False-color SEM image of the experimental device. (c, d) XMCD contrast images utilizing Co L_3_-edge and Fe L_3_-edge excitations to visualize the magnetization in the Co and Py structures, respectively. (e) Comparative analysis of magnetic contrast between an isolated Co bar, a Co bar enclosed within the magnetic flux concentrator (MFC) and the MFC central core for increasing externally applied magnetic fields oriented perpendicular to the bar. (f) Micromagnetic simulations of the experiments conducted in (e). (g, h) The evolution of the average magnetic contrast as a function of the applied field is depicted for experimental (g) and simulated (h) data. This includes the isolated Co bar (Co), the Co bar within the MFC (Co + MFC), and the MFC itself. Dashed lines outline the regions where signal averaging is performed in panels (c, d). The inset of panel (g) compares XMCD contrast and the SQUID signal obtained from an array of Co bars with identical dimensions.

The magnetic responses of both the Py flower-shaped metasurfaces and the inner Co bar were imaged at room temperature by means of XPEEM using X-ray Magnetic Circular Dichroism (XMCD) as a magnetic contrast mechanism (see Figure 2(a) and Methods). Measurements were conducted on the central region of a 10-petal flower-shaped metasurface with inner and outer radii of *R*_i_ = 10 μm and *R*_0_ = 100 μm, respectively, and an inner slot of width 4 μm. A Co bar 20 μm long, and 5 μm wide was nanofabricated and positioned at the slot’s center, slightly overlapping to ensure no gap between them. The thickness of the Co bar, *t*_Co_ = 50 nm, is slightly less than that of the Py MFC, *t*_Py_ = 60 nm. This difference in thickness permits the isolation of the 4 μm-wide bar inside the gap from the overlapping Co, as evidenced by pink lips in the surface topography image in Figure 2(b). Images were also acquired on a reference isolated bar to evaluate the effect of the MFC. Figure 2(c,d) show the magnetic contrast XMCD images of the Py metasurface and Co bar obtained at the Fe *L*_3_-edge and Co *L*_3_-edge, respectively. Interestingly, the Co nanowire formed by the overlapping of the Co and the Py deposited patterns does not show any significant XMCD signal. XMCD images were acquired as a function of the externally applied in-plane magnetic field perpendicular to the bar long axis for fields ranging between 0 and 10 mT after magnetic saturation at −100 mT. XMCD maps at selected external magnetic fields are presented in Figure 2(e) for the isolated reference Co bar, the bar inside the MFC, as well as the central core of the MFC. These XMCD maps reveal an alternating contrast in the isolated bar, suggesting the presence of multiple magnetic domains. As the magnetic field increases from 0 to 10 mT, the domains oriented along the applied field slowly grow, but the bar remains unsaturated.

In contrast, the magnetic signal of the Co bar surrounded by the MFC reverses abruptly from negative to positive saturation at 2.5 mT, correlating with the preferential magnetization direction observed in the Py MFC. This behavior is well replicated in micromagnetic simulations, as demonstrated in Figure 2(f). There is, however, a quantitative discrepancy of a factor two in the magnetic field at which the magnetization switching occurs. This can be attributed to the size differences between the experimental sample and the simulated one, implying different demagnetization fields (see Tables 1 and 2). The above observations naturally suggest that the effective coercive field and susceptibility of the Co bar can be tailored by modifying the geometry of the used metasurface. This point will be examined in detail in the following section. It should be noted that there is a slight difference between the experimental and simulated magnetic domain distributions. This difference can be attributed to defects in the magnetic films induced by the nanofabrication process. For instance, edge roughness or point defects in the granular structure of the thin film can lead to the emergence of additional pinning sites, resulting in variations in the domain arrangement within the device.

**Table 2:** Dimensions of Co Sensor Bars (Thickness, *t*_Co_, Width, *w*, and Length, *L* = 2 *R*_i_) and Py Metasurfaces (Inner Ratio, *R*_i_, Radii Ratio, *r* = *R*_o_/*R*_i_, Thickness, *t*_Py_, Gap *d*_gap_, and Number of Petals, *N*_p_) Used for Simulations

figure	*t*_Co_ (nm)	*w* (μm)	*R*_i_ (μm)	*r* = *R*_0_/*R*_i_	*t*_Py_ (nm)	*d*_gap_ (nm)	*N*_p_
Figure 2f,h	30	0.8	2	3	50	0	10
Figure 3d,e	30	0.8	2	3	50	30	10
Figure 6a,d	30	0.8	2	1.25–4	50	3	10
Figure 6b,e	30	0.8	2	3	50	20–200	10
Figure 6c,f	30	0.8	2	3	50	30	2–26
Figure 8e–f	60	0.1–2.5	0.2–2	3	100	30	10

A more insightful representation of the magnetization switching process can be achieved by analyzing the average contrast within a specific region (outlined by dashed contours in Figure 2(c,d)) as a function of the applied magnetic field. This analysis is presented in Figure 2(g) for XPEEM results and Figure 2(h) for the micromagnetic simulation. The abrupt reversal of magnetization in the Co bar surrounded by the MFC (Bar + MFC) is prominently observed between 2 and 2.5 mT in the experimental data and between 6.25 and 7.5 mT in the simulations. Both hysteresis curves exhibit qualitative similarities, with the sharp magnetization reversal in the Co bar occurring concurrently with the magnetization reversal of the MFC central core. This is a key finding of this work, demonstrating that the coercive field of the Co bar is fully controlled by the MFC. In contrast, the reference isolated bar displays a smooth linear change in magnetization, a behavior typically associated with a ferromagnetic bar when subjected to a magnetic field aligned with its (short) hard axis (HA).^36^ This observation is consistent with the results obtained from the Superconducting Quantum Interference Device (SQUID) measurement on an array of identical Co bars, as depicted in the inset of Figure 2(g).

### Planar Hall Co Bar Magnetic Sensors

**Figure 3:**
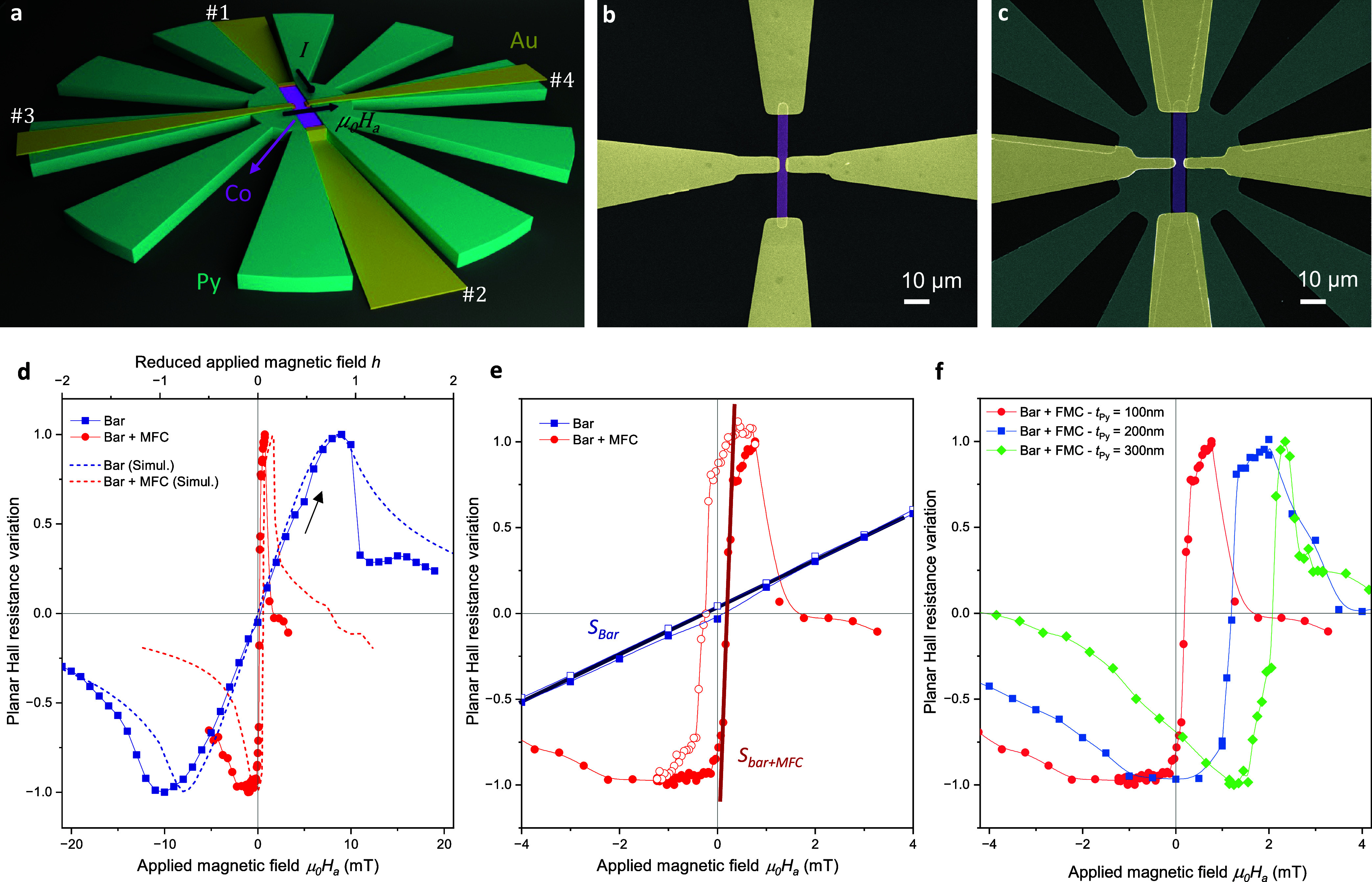
(a) Schematic illustration of a magnetic metasurface indicating the contact geometry used for PHE measurements. Current is injected from electrode #1 to #2 while transverse Hall voltage is measured using electrodes #3 and #4. Arrows show the current and magnetic field direction. (b, c) False-color SEM images of a reference Co bar (b) and a Co bar inside a metasurface (c). (d) Experimental variation of the normalized planar Hall resistance as a function of the applied magnetic field (bottom abscissa) obtained for a reference Co bar (blue dots) and a bar placed inside a 10 petal metasurface of *t* = 100 nm (red dots). The arrow indicates the magnetic field sweep direction. Dashed lines show the micromagnetic simulations plotted as a function of the reduced applied field (top abscissa). (e) Magnification of the experimental curves shown in (d) with the values *S*_bar_ and *S*_bar+MFC_ indicated. Closed and open points show up and down magnetic field sweeps, respectively. (f) Field dependence of the planar Hall resistance measured for identical Co sensor bars placed inside metasurfaces of different thicknesses. Red dots correspond to the experimental curve shown in (d, e).

Concentrators with various geometries have been designed to assess the potential for tuning the magnetic response of Co bars. As depicted in Figure 3(a), these concentrators feature a central Co bar sensor connected to Au electrodes in a transverse configuration to perform Planar Hall Effect (PHE) measurements.

This configuration offers a high signal-to-noise ratio with sensitivity to the direction of magnetization, making it a well-suited technique for highlighting the influence of magnetic metasurfaces on the Co bar magnetization reversal.^37^ An insulating layer is deposited on top of the Py MFC before the deposition of Co and Au structures to prevent current leakage. It is important to note that the insulator enforces a minimal distance between both ferromagnetic layers, further ensuring no magnetic exchange interaction at the interface. As a reference, identical isolated Co bars were also fabricated for comparison. SEM images of the final devices are shown in Figure 3(b,c). In our setup, a DC current is injected into the Co bar via electrodes #1 and #2, and a transverse voltage signal resulting from spin-dependent scattering phenomena is probed between electrodes #3 and #4. The measured transverse resistance depends on the angle φ between the directions of magnetization and the charge current flow:
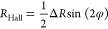
1

where Δ*R* represents the difference in longitudinal resistance between magnetization parallel and perpendicular to the current flow. PHE measurements may exhibit two distinct characteristic signals. In the simplest scenario, the magnetization of the sensing bar resembles that of a Stoner–Wohlfarth particle,^38^ displaying a single domain that rotates based on the direction and magnitude of the external magnetic field. In this case, the PHE signal, *R*_Hall_, follows a typical sinusoidal shape with a linear response at the low applied field and two extrema when the magnetization direction forms an angle φ = ±45° with the applied current. In contrast, when the bar contains a multidomain structure with diamond or Landau-pattern arrangements, the local magnetization direction may consistently remain parallel or perpendicular to the direction of current flow, thus leading to minimal changes in the transversal resistance. Consequently, high aspect ratio Co bars were utilized to favor a single-domain configuration (see Tables 1 and 2).

Figure 3(d) illustrates the variation of the planar Hall resistance as a function of the applied field for both an isolated bar of *t*_Co_ = 60 nm, *w* = 5 μm, *L* = 50 μm (blue squares) and an identical bar positioned within a metasurface featuring 10 petals with *R*_i_ = 25 μm, *R*_0_ = 125 μm, and *t*_Py_ = 100 nm (red dots). Both curves exhibit the anticipated PHE behavior with a linear dependence around zero magnetic field and maximum or minimum values, attributed to an averaged magnetization forming angles φ = ± 45° with the current direction. It is important to note that the metasurface induces a high field concentration effect, leading to a significant enhancement of the linear slope and a reduction in the peak-to-peak distance. These effects show a substantial increase in the sensitivity of the Co bar sensor, which we characterize through the Hall resistance slope as *S* = δ*R*_Hall_/δ*H*_a_, being *S*_bar+MFC_ and *S*_bar_ the sensitivity of the sensor within the metasurface and the reference one, respectively (see Figure 3(e)). Moreover, in addition to the flux concentration effect, the presence of the MFC leads to the opening of the hysteresis in the Co bar, as illustrated in Figure 3(e), showing a zoom at a low applied magnetic field of the planar Hall variation obtained with positive (closed symbols) and negative (open symbols) magnetic sweeps. This hysteresis would determine the sensor operating point, H*, defined as the point where the Hall resistance crosses zero.

Micromagnetic simulations were conducted to replicate the PHE measurements, assuming a uniform current density distribution throughout the entire Co bar. Simulated curves are represented by dashed lines in Figure 3(d), where the normalized planar Hall resistance signal of a 30 nm thick, 4 μm long, and 800 nm wide Co bar, surrounded by a Py MFC with *R*_0_/*R*_i_ = 3 (red dashed lines) is compared with an isolated one (blue dashed lines). Despite differences in dimensions, the simulated behavior of the Co bar closely reproduces the experimental variation trends. A qualitative comparison is facilitated by introducing a reduced magnetic field, *h*, calculated as the Co bar’s ratio between Zeeman and shape anisotropy energy
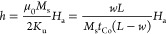
2

where  is the shape anisotropy energy, *N*_HA_ and *N*_EA_ are the hard axis and easy axis demagnetization factors calculated based on the projected surface areas,^39^*M*_s_ is the saturation magnetization, and *t*_Co_, *L* = 2*R*_i_, and *w* are the thickness, length and width of the Co bar, respectively. The slight differences observed between the experimental and simulated curves could be attributed to the presence of pinning centers leading to an abrupt change in signal, a misalignment of the applied field, a difference between experimental and simulated electrode position, or a dependence on the permalloy properties and quality (via the Brown paradox^40^). These factors cannot be straightforwardly addressed by considering a reduced applied field based on the shape anisotropy. However, it is worth noting that, first, both the experiment and simulation exhibit the same trend regarding the Hall resistance hysteresis and slope. Second, the differences observed suggest the possibility of controlling the sensor operating point, H*, and/or the variation rate of the sensitivity, *S*_bar+MFC_/*S*_bar_, by tuning the MFC geometry. For instance, an increase in the thickness of the MFC results in an increase of the demagnetization factor of the MFC, leading to a substantial increase in H*, as depicted in Figure 3(f). This figure provides a close-up view around zero field of the experimental curve obtained for the bar inside the MFC shown in Figure 3(d), compared with two other bars surrounded by metasurfaces of identical dimensions but different thicknesses. It is noteworthy that the absence of direct proportionality between H* and the thickness of the MFC (as anticipated from shape anisotropy) is due to the abrupt increase of coercive field of Py thin films with thickness larger than 120 nm as shown in Supporting Information (Figure S1), attributed to the tendency of Py to develop a stripe-like magnetic texture for thicknesses above 100–200 nm.^41^

### Tuning the Performance of Co Bar Magnetic Sensors

**Figure 4:**
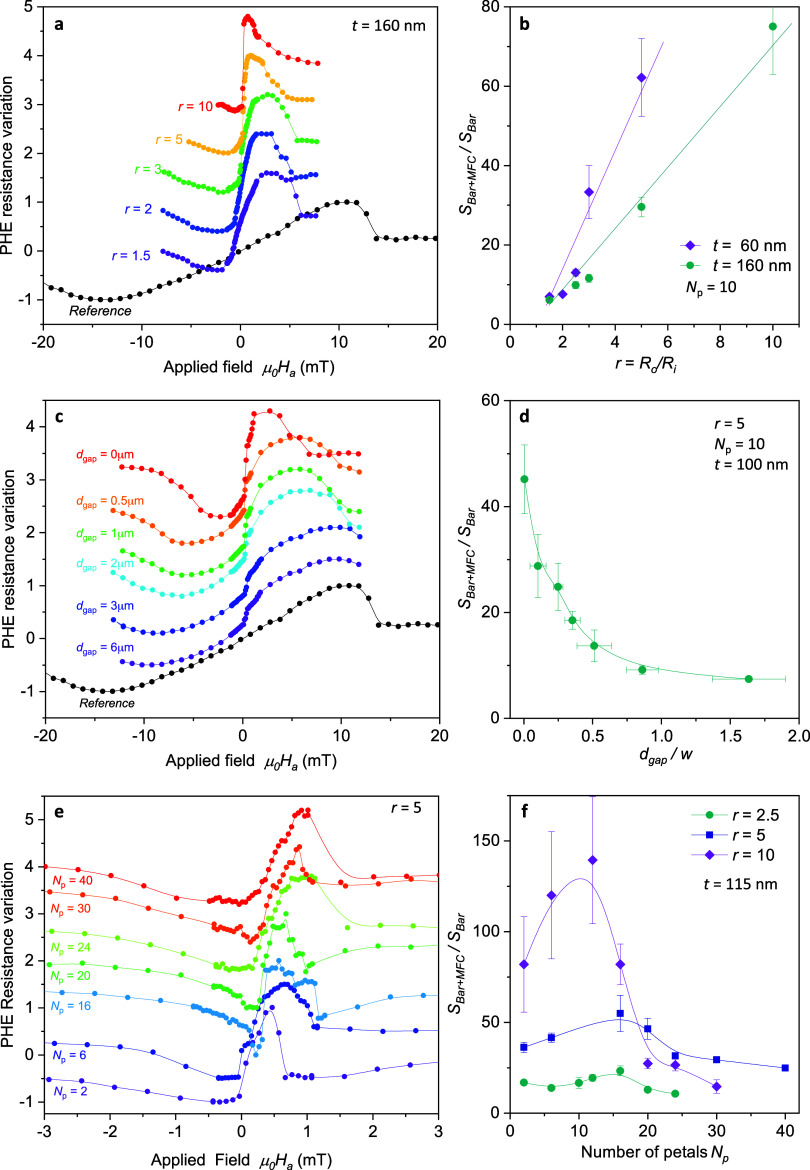
Experimental measurements of the planar Hall resistance variation as a function of the applied magnetic field (curves are vertically shifted for clarity) and the values of *S*_bar+MFC_/*S*_bar_ obtained for a series of devices with different *r* = *R*_o_/*R*_i_ (a, b), *d*_gap_ (c, d), and number of petals (e, f). The dimensions of all the metasurfaces indicated in the panels are summarized in Table 1.

**Figure 5:**
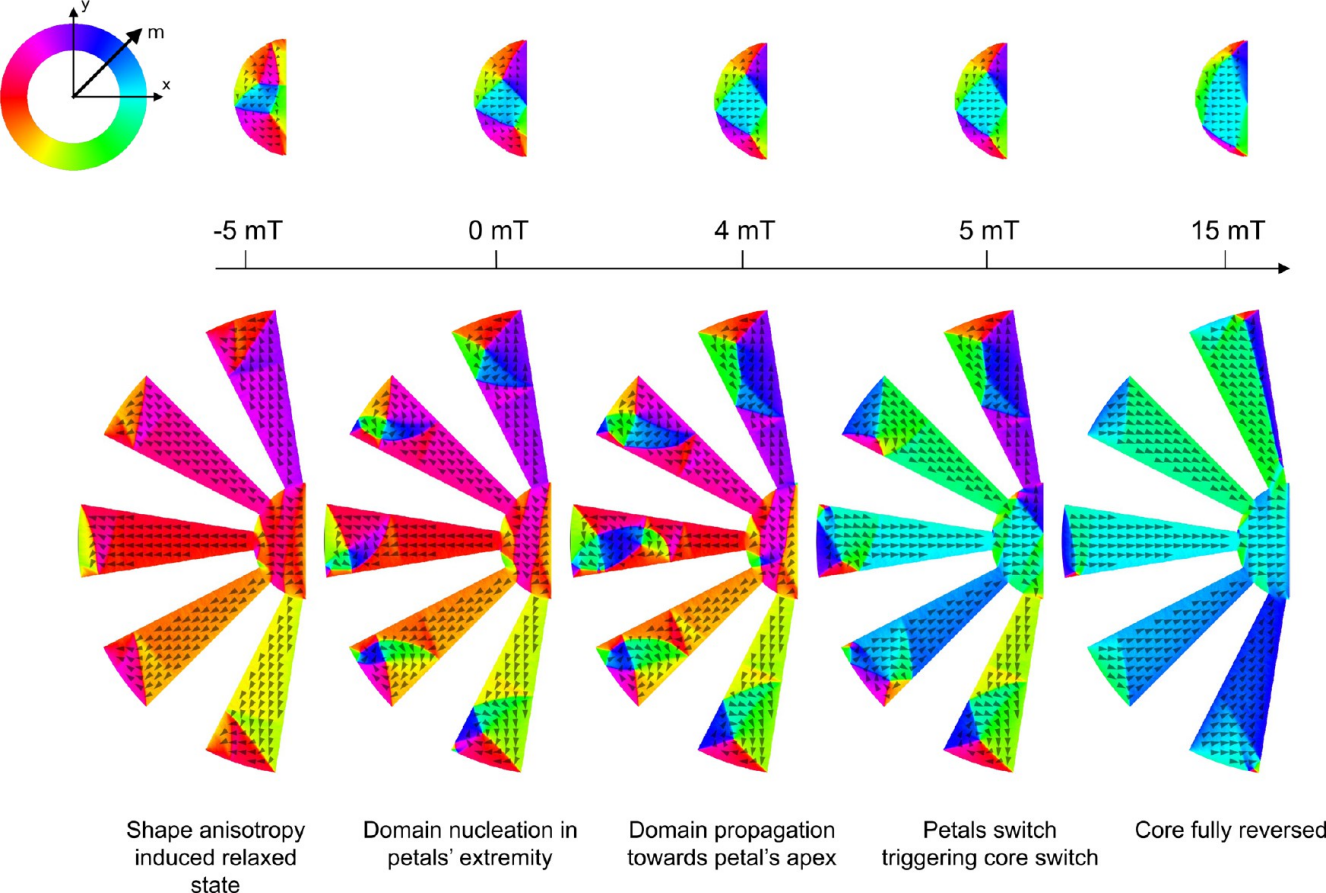
Micromagnetic simulations showing the mechanisms governing the magnetic field concentration in a 10-petal metasurface *R*_0_/*R*_i_ 3.5. For the sake of clarity, only the left part of the device is shown (the right part being essentially a mirror version of domain distribution). Top panels correspond to the magnetic domain distribution in an MFC without petals, serving as a reference and permitting us to understand the mechanisms in action in the presence of petals (bottom panels). The magnitude of the applied field corresponds to the projection along the *x*-axis.

In the following, we assess the impact of various geometric parameters of the magnetic metasurfaces on both the sensitivity and operating point of the sensing bars. Figure 4(a,c,e) show the normalized Hall resistance plotted against the applied field for a series of devices with a constant inner radius *R*_i_ = 25 μm and radius ratio *r* = *R*_0_/*R*_i_, gap distance *d*_gap_, and number of petals, *N*_p_, respectively (see Table 1 for dimensions). Curves are vertically shifted for clarity. Figure 4(b,d,f) show the variation of bar sensor sensitivity, *S*_bar+MFC_/*S*_bar_, obtained for Co bars placed inside the different metasurfaces where clear trends with their geometry are observed, as will be discussed in the following. Note that a dramatic enhancement in *S*_bar+MFC_/*S*_bar_ by more than 2 orders of magnitude may be obtained, evidencing the large magnetic field concentration effect that can be achieved by combining the metamaterial with the thin film demagnetizing effect.^35^ The mechanism governing the magnetic flux concentration within the metasurfaces is illustrated in Figure 5. Initially, the system is fully saturated with a large magnetic field in magnitude directed toward the left. Subsequent reduction of the applied field intensity causes the shape anisotropy of the petals to compel domain alignment along the petals’ EA, thereby influencing the orientation of magnetic domains in the central core, which remain firmly pinned in the direction of the applied field. For comparison, the distribution of magnetic domains in an isolated core is also presented (top panels). As the applied magnetic field varies, vortices nucleate at the extremities of the petals (where the aspect ratio is lowest) and propagate toward the apex of the petals and the central core, which predominantly maintains its magnetization in the direction opposite to the applied field. Further increasing the applied field will induce magnetic reversal in the petals, triggering a reversal process in the core, resulting in an amplified concentrated stray field within the metasurface gap.

**Figure 6:**
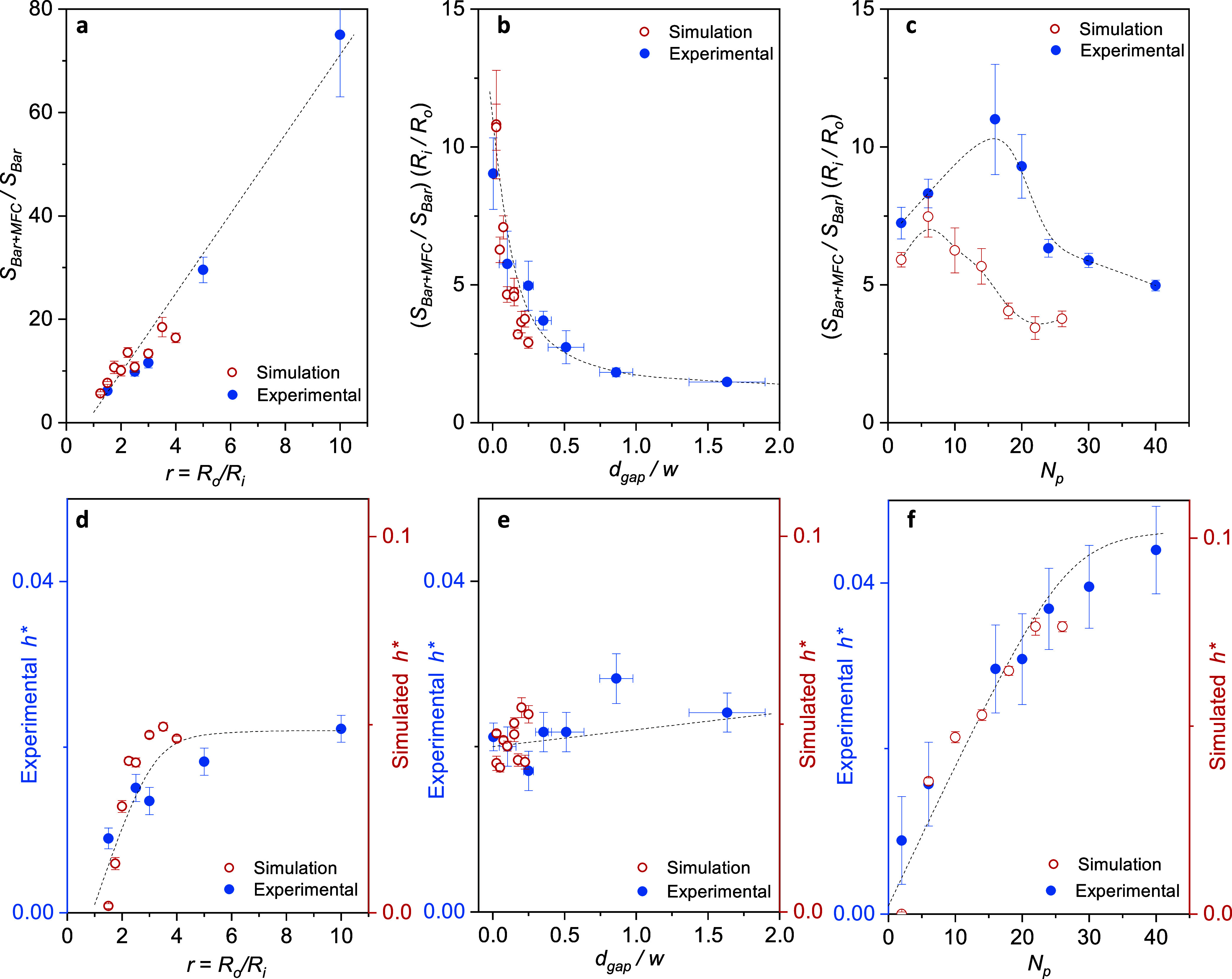
Experimental (solid blue dots) and simulated (open red dots) values of *S*_bar+MFC_/*S*_bar_ and h* obtained for a series of devices with different *r* = *R*_*o*_/*R*_*i*_ (a, d), *d*_gap_ (b, e), and number of petals (c, f). The values of *S*_bar+MFC_/*S*_bar_ in (b,c) have been normalized by *R*_*i*_/*R*_*o*_. Dashed lines are guides to the eye. The dimensions of experimental and simulated metasurfaces are summarized in Tables 1 and 2, respectively.

Figure 6 shows the trends of the sensitivity enhancement and sensor operating field as a function of the different metasurface parameters analyzed (*R*_0_/*R*_i_, *d*_gap_, *N*_p_) obtained from both experimental measurements and micromagnetic simulations. For a qualitative comparison between experimental data and simulations we plot (*S*_bar+MFC_/*S*_bar_) × (*R*_i_/*R*_0_) in Figure 6(b,c) and the reduced operating magnetic field, h*, in Figure 6(d–f). Figure 6(a,d) shows the evolution of *S*_bar+MFC_/*S*_bar_ and h* with *r* = *R*_0_/*R*_i_. A linear trend of the sensitivity enhancement is evident in both the experimental device (filled blue dots) and the modeled ones (empty red dots) consistent with flower-shaped metasurface responses reported in the literature.^30^ In the case of h*, the primary trend indicates that the presence of petals results in a higher switching field by anchoring the magnetic domains at different locations in the core.

Additionally, a linear increase in the switching field with the length of the petals is observed for lower values of *r* until it tends to saturate for larger radii ratios. This behavior is attributed to the interplay between the petals and the central core of the device. In the case of small petals, the core dominates the switching field, leading to a low coercive field due to the absence of a preferential in-plane (IP) magnetization axis. In this regime where *R*_0_ ≃ *R*_i_, the magnetic domains in the petals impose little constraint on the core, which tends to relax into a vortex state due to the low IP shape anisotropy (the petal is as wide as it is long), limiting their impact on the domain distribution in the core. As the outer radius *R*_0_ increases, both the length of the petals and the outer width (far from the core) increase, while the inner width remains constant. Consequently, magnetic domains have more freedom to rearrange at the extremity of the petals to reduce the magnetostatic energy as the external field varies without compelling the rotation of the domains near the core, thus delaying the magnetic switching. However, the local relaxation starting from the petal’s outer extremity becomes unstable for large petals. It leads to a sudden magnetization reversal (buckling domains) of the petal, with the propagation of the domain walls occurring in a narrow range of magnetic fields, triggering the switch of the central core and then the Co sensor. This is better elucidated through micromagnetic simulations (Supporting Information, Figure S2), showing that magnetic reversal occurs simultaneously in the petals and the core in long-petals devices. In contrast, it occurs first in the central core for short petals. It is worth noting that a different *y*-axis scale has been used for simulated and experimental results, for which the difference is attributed to Brown’s paradox as explained above. Figure 6(b,e) present the dependence with the gap separating the edges of the Py MFC and the sensing Co bar. Results show a decrease in sensitivity enhancement with an increasing gap, which can be attributed to the reduction of the dipolar field intensity with distance. Regarding the switching field, no significant variation is observed, confirming the hypothesis that the Co sensor does not influence the domain switching inside the Py metasurface. Finally, Figure 6(c,f) depict the effect of the number of metasurface petals. The optimal concentration effect is obtained for the thin film flower-shaped metasurface with a petal angle equal to the gap between adjacent petals.^33^ So, an increase in the number of petals goes hand in hand with a reduction of the petal’s width, leading to an increase of the shape anisotropy energy of the petals parallel to the applied field (those which contribute the most) and then a higher switching field of the device, as presented in Figure 6(f). Figure 6(c) presents an intriguing observation indicating that the sensor sensitivity enhancement exhibits a peak, the position of which depends on the dimensions of the MFC (see also Figure 4(f)). This phenomenon is ascribed to the interplay between two factors that alter the petals’ width. On the one hand, the effective permeability of an individual petal increases with its aspect ratio. Thus, a greater value of *N*_p_ results in a more sudden (i.e., occurring on a smaller range of applied field) propagation of domain walls from the petal extremity (where they nucleate) toward the petal’s apex and the core, forcing the magnetic switch of the latter. Consequently, an increase of the sensitivity with *N*_p_ is observed, in agreement with theoretical predictions,^30^ albeit the mechanism may not be entirely analogous (see Supporting Information, Figure S3). On the other hand, narrower petals exert a diminished influence on the central core, meaning that the magnetic switching of a single petal is insufficient to fully reverse the magnetization in the core. This insight, combined with the fact that the switching field of each petal depends on the orientation of its EA relative to the applied field direction, implies that the core undergoes a smoother, step-by-step magnetic reversal process. Instead of the abrupt switch triggered by the reversal of a single wide dominant petal (aligned with the applied field), the Py core experiences a gradual penetration of domain walls, leading to a more gradual switching process and, consequently, a reduction in sensitivity (this effect is illustrated in the Supporting Information based on micromagnetic simulations). Additionally, as it is directly related to the aspect ratio of the petals, the optimal value of the sensitivity enhancement is expected to vary with the device geometry. Specifically, longer petals have a greater impact on the core, leading to an efficiency peak for a larger number of petals in devices with a greater ratio *R*_0_/*R*_i_. Similarly, devices composed of shorter petals should be less affected by the number of petals.

### Substructured Metasurfaces

We have demonstrated that the metasurface geometrical parameters (thickness, radii, petals, gap) can be adjusted to tune both the sensitivity and operating point of magnetoresistance sensors. The ability to control the effective coercive field, H*, of magnetic structures holds significant promise for applications based on magnetization reversal processes, which can be obtained at extremely small magnetic fields, *H* < H*. However, for the particular case of magnetic sensors, this means that the sensor must be magnetically biased to this operating point, which may be regarded as an impractical aspect of the device. A possible way to bring the operating point closer to zero field would be to use even softer ferromagnetic materials or superparamagnetic nanostructures. An appealing and elegant alternative solution consists of tuning the magnetic response of the petals by substructuring them so that the direction of easy magnetization of each substructure is perpendicular to the direction of the magnetic flux lines.

**Figure 7:**
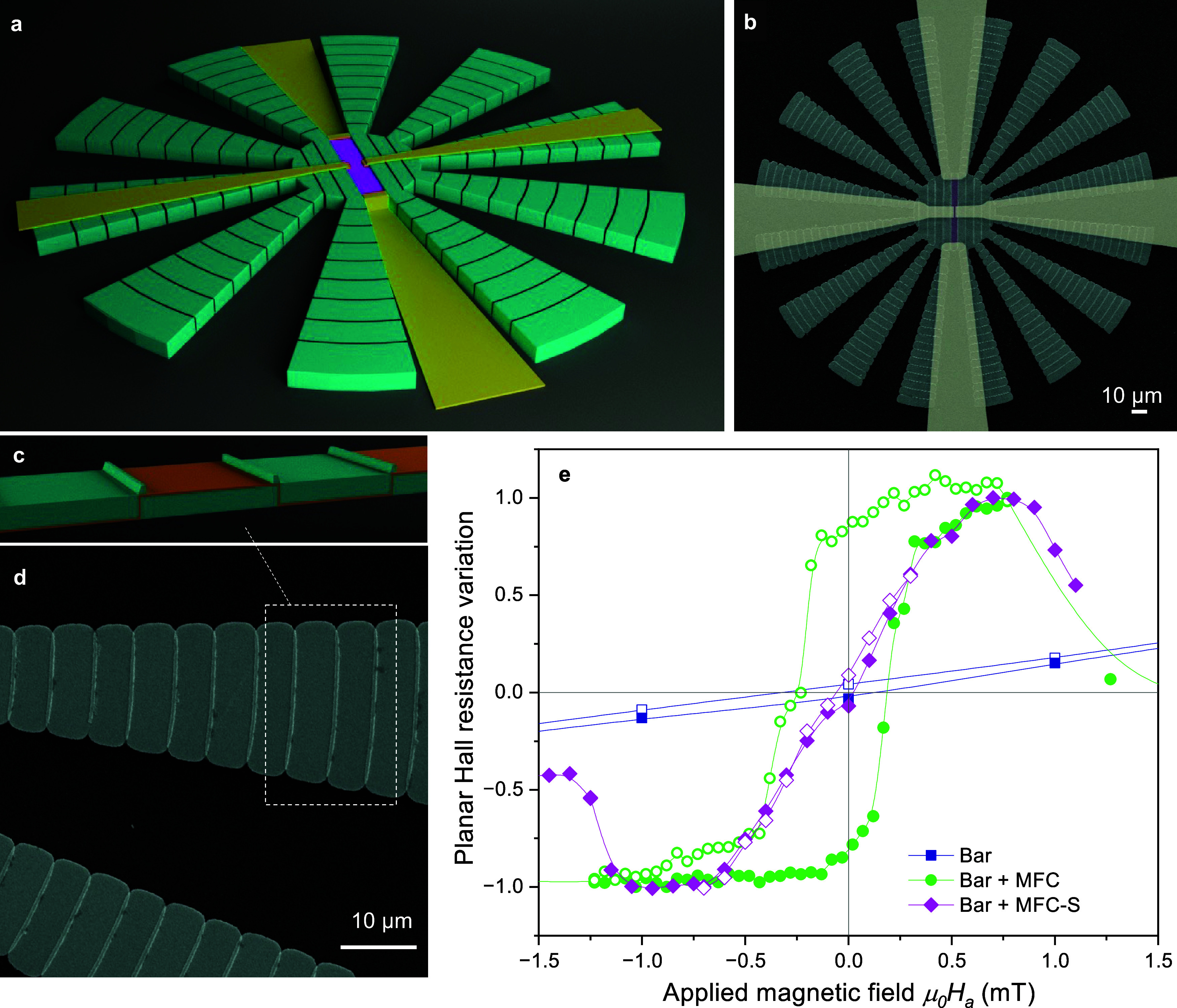
(a) Schematic illustration of a Co bar sensor located inside a magnetic metasurface with segmented petals. (b) False-color SEM image of the full device. (c) Schematic representation of a petal cross-section. (d) SEM image of a magnified petal. (e) Experimental variation of the normalized planar Hall resistance as a function of the applied magnetic field obtained for a reference Co bar (blue), a bar placed inside a 10-petal metasurface with unpatterned petals (green) and segmented petals (magenta). Closed and open points show increasing and decreasing magnetic field sweeps, respectively.

Figure 7(a,b) illustrate a schematic representation and SEM image, respectively, of a metasurface featuring substructured petals that are transversely segmented into small pieces. A magnetic insulating spacer has been introduced between the slightly overlapping pieces to optimize the concentration effect and prevent magnetic coupling between substructures, as illustrated in Figure 7(c,d). Figure 7(e) shows the variation of the planar Hall resistance measured for a Co sensor bar located inside the metasurface made of segmented petals, compared with the data obtained for a metasurface with nonpatterned petals and a reference sensor. It can be observed that the metasurface with segmented petals strongly concentrates the magnetic field, inducing an enhancement of the sensor sensitivity with an operating point at zero applied field and a reversible slope. However, it should be noted that the sensitivity of the bar for this particular metasurface geometry is reduced from (*S*_bar+MFC_/*S*_bar_) ≃ 50 to 15 with the patterning. There is room for optimizing this value by tuning the metasurface’s geometrical parameters and patterning of petals through tuning the dimensions of the substructures and the nonmagnetic spacer.

### Miniaturization of Metasurfaces for On-Chip Applications

**Figure 8:**
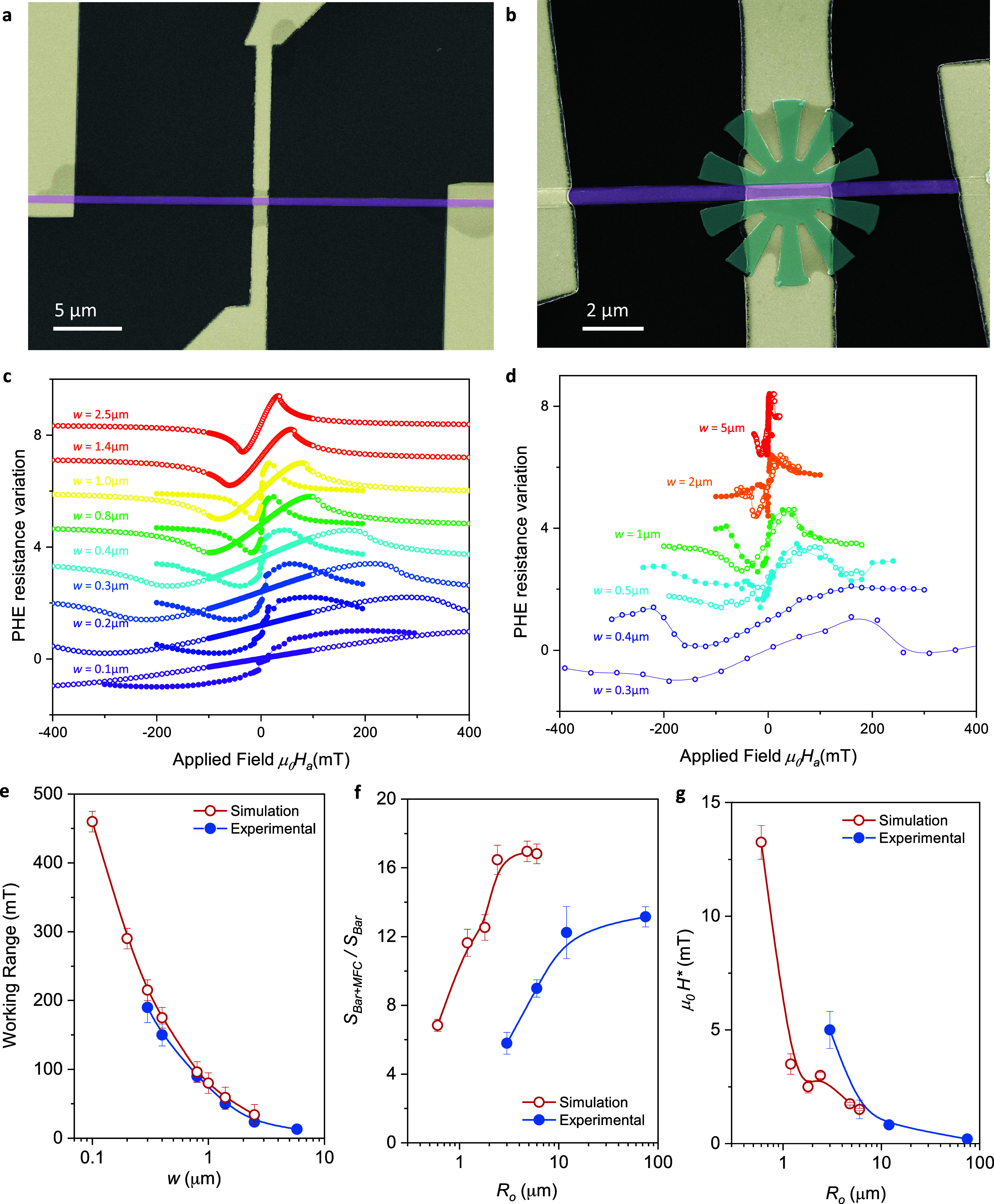
(a, b) SEM image of an isolated and a Co bar sensor with *w* = 300 nm and a bar of the same width located inside a magnetic metasurface. (c, d) Simulated and experimental variation of the normalized planar Hall resistance measured at the center of the bar as a function of the applied magnetic field obtained for reference Co bars of different widths (open symbols) and same bars placed inside a 10-petal metasurface of *R*_0_ = 6*w*. (e–g) Experimental (solid blue dots) and simulated (open red dots) values of the working range, *S*_bar+MFC_/*S*_bar_ and H* obtained for a series of devices with different Co rod widths.

Let us now evaluate the scalability of metasurfaces down to the mesoscopic scale. Our goal is to identify the limits of the miniaturization of metamaterial structures and to evaluate their performance as the size of the petals approaches the scale of magnetic domains. Figure 8(a,b) present SEM images of a 500 nm wide reference Co rod sensor and a rod sensor integrated within a metasurface of external radius, *R*_*o*_ = 3 μm and internal radius, *R*_*i*_ = 1 μm. The variation of the planar Hall resistance as a function of the applied magnetic field has been investigated for a series of devices with progressively scaled down dimensions (the ratio between relevant geometrical parameters of each device is maintained constant, except for the gap *d*_gap_ and the thicknesses *t*_Co_ and *t*_Py_). Results obtained from micromagnetic simulations and experimental measurements are reported in Figure 8(c,d), respectively, comparing the isolated reference Co rod (open symbols) and the Co rod within a metasurface (closed symbols). More details on the dimensions of the devices are provided in Tables 1 and 2.

Figure 8(e) shows that the linear magnetic range for the isolated rod expands as the rod width is reduced, which is attributed to the increased demagnetization factor for the configuration used in the present experiment. Numerical simulations quantitatively confirm this trend. When considering the impact of the Py MFC, a decrease in the concentration gain, *S*_bar+MFC_/*S*_bar_ and an increase in the coercive field of the Co rod are observed as the device is scaled down, with a consistent trend in both micromagnetic simulations and experiments. This result is due to the fact that a smaller MFC corresponds to less magnetic material and, consequently, a lower variation of the bar sensor sensitivity. Similarly, the saturation of *S*_bar+MFC_/*S*_bar_ for larger devices is explained by the rapid decrease in magnetic dipolar interactions with distance, implying that the additional ferromagnetic material has less impact on larger devices. Other parameters controlling the sensor sensitivity, highlighted in the previous section, such as the gap, the number of petals, and the radii ratio, are kept constant. The quantitative discrepancy between simulations and experiments for the *S*_bar+MFC_/*S*_bar_ values presented in Figure 8(f), is attributed to a larger gap in the experimental devices. Specifically, lithography accuracy, possible oxidation of both Co and Py, and the presence of magnetic dead layers result in an actual gap larger than the targeted value of 30 nm. Indeed, as shown in Figure 6(b), the variation of the bar sensitivity is highly dependent to the size of the gap, particularly for very small gaps.

**Figure 9:**
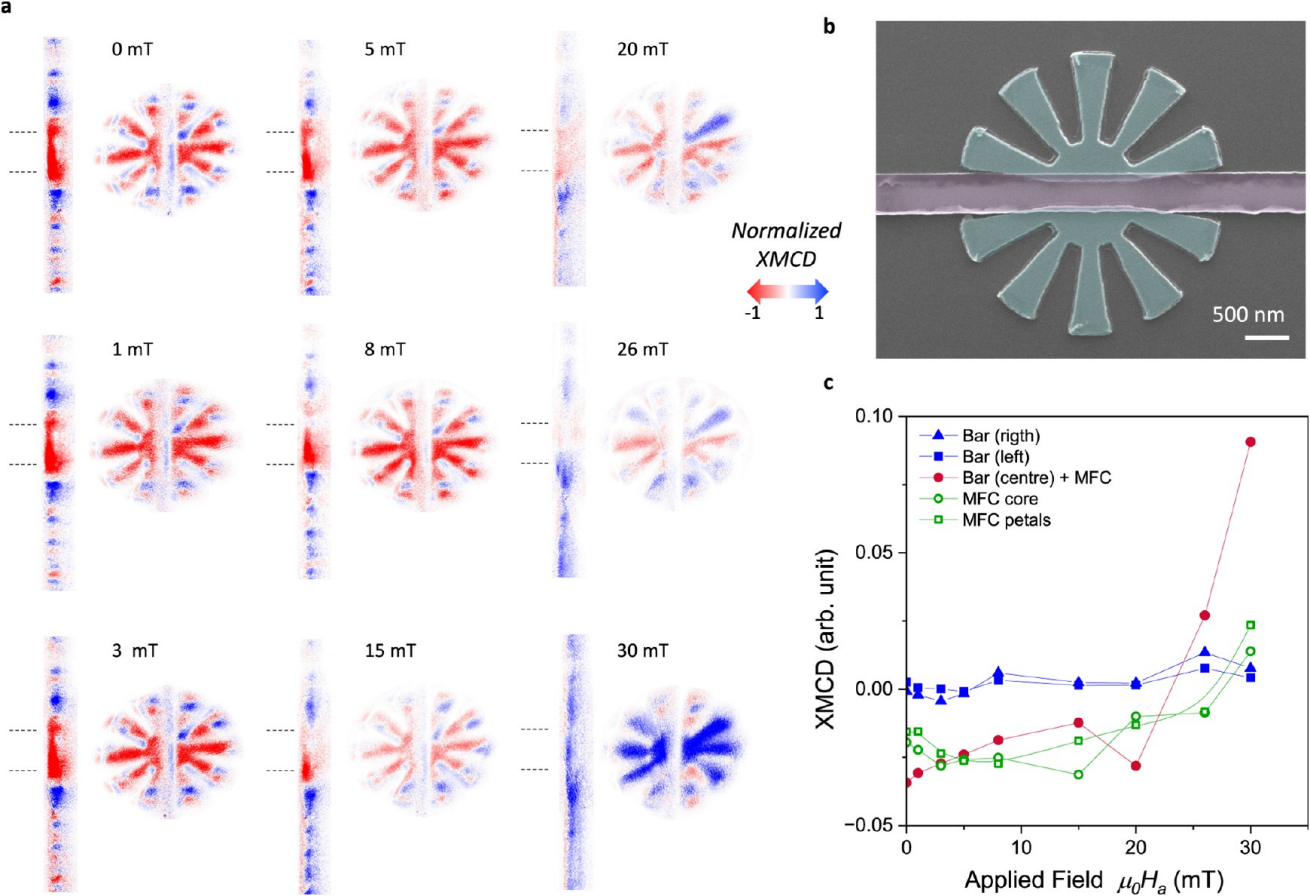
(a) Analysis of the magnetic contrast of a Co bar of width *w* = 400 nm and length 6 μm, enclosed within a metasurface of *R*_o_ = 1.5 μm and *R*_i_ = 500 nm for increasing externally applied magnetic fields oriented perpendicular to the bar. Dashed lines indicate the position of the metasurface. (b) False-color SEM image of the central part of the Co bar inserted within the metasurface. (c) Evolution of the average magnetic contrast as a function of the applied field obtained for the Co rod at different regions outside (left and right) and inside of the MFC (center), as well as for the MFC core and petals.

To elucidate the behavior of the metasurface as its petals approach the size of the magnetic domains, we present in Figure 9(a) magnetic contrast XMCD images of a mesoscopic Py metasurface with *t* = 60 nm, *R*_*o*_ = 1.5 μm, *R*_*i*_ = 500 nm, and a 400 nm wide, 6 μm long Co rod located within the metasurface. Figure 9(b) shows a SEM image of the device. The magnetic images were acquired while varying the externally applied in-plane magnetic field perpendicular to the EA of the Co bar, for fields ranging from 0 to 30 mT after magnetic saturation at −100 mT. One can clearly see that, due to the magnetic field concentration within the metasurface, the magnetic domains in the Co bar can be completely reversed within this region, while the region of the Co bar lying outside the concentrator remains unsaturated. Figure 9(c) displays the average contrast of the magnetization of the Co bar both inside and outside the metasurface as a function of the applied magnetic field and for the MFC core and petals. As highlighted in the case of larger metasurfaces, the abrupt reversal of the magnetization in the Co bar located within the MFC occurs simultaneously with the magnetization reversal of the MFC central core. As experimentally observed in Figure 9(a), the magnetization of each petal is progressively subject to a sudden reversal as the applied field increases, depending on the orientation of their EA, except for petals with an EA perpendicular to the applied field where a multidomain structure is formed. The full magnetization reversal of the FM core of the metasurface is in turn triggered by the switch of the last petal. An important variation in the Hall resistance slope is obtained *S*_bar+MFC_/*S_b_*_ar_ = 32, clearly indicating that the effect of the concentration of the metasurfaces is preserved even when the dimensions are significantly reduced.

## Conclusions

In conclusion, this work presents a pioneering method for integrating on-chip magnetic metasurfaces into magnetoresistance sensing devices, resulting in improved performance. Our experimental and theoretical results demonstrate that by appropriately selecting the metasurface geometrical parameters, we can tune the response of magnetoresistance Co bar sensors to obtain precise control of the coercive field and susceptibility. One significant technical advancement achieved is the substantial increase in magnetoresistance sensor sensitivity by over 2 orders of magnitude. This improvement can be further optimized by adjusting the metasurface’s size, as there is a direct correlation between the enhancement of sensitivity and the increase in the metasurface radii ratio. Moreover, we show that patterning the metasurface petals may provide a new “knob” for controlling the properties of the metasurface. The scalability of the device has been validated by analyzing the performance of the metasurfaces as they approach the size of magnetic domains. These results highlight the potential of magnetic metasurfaces, which may be exploited to improve the performance of magnetic sensors or add new functionalities to other magnetic devices. In particular, they offer the possibility to control the coercive field and saturation of magnetic structures at the very local scale without changing their magnetic anisotropy. This opens up new opportunities for integrating metasurfaces into functional spintronic devices or improving the efficiency of small-scale magnetic energy harvesting materials. Moreover, a concrete application consisting of saturating small-volume magnetic micro- and nanostructures by applying extremely low magnetic fields may benefit magnetic characterization tools using high-resolution techniques unable to cope with large magnetic fields.^42^

## Methods

### Experimental Section

Py flower-shaped Permalloy (Py) metasurfaces and Cobalt (Co) bars were fabricated by sputtering on Si substrates, using photolithography or Electron Beam Lithography (EBL) and lift-off. For the Planar Hall experiments, the Py structures were covered by a 30 nm-thick Al_2_O_3_ layer grown by Atomic Layer Deposition before the growth of Co bars, and 50 nm-thick Au contacts were patterned on top by sputtering and lift-off. Py structures of different dimensions; internal ratio (*R*_i_), external ratio (*R*_0_), thickness (*t*_Py_), number of petals (*N*_p_), and gap (*d*_gap_) were fabricated. A Co bar of length 2*R*_i_, width *w*, and thickness *t*_Co_ was placed inside the metasurface. Reference isolated Co bars were grown for comparison. Table 1 summarizes the dimensions of all the samples analyzed.

For the Planar Hall measurements (see Figure 3(a)), a constant current of *I* = 10 mA was injected along the EA from electrode #1 to #2, while the resulting transverse voltage change was monitored through electrodes #3 and #4. The measurement is repeated with various external magnetic fields applied in the plane of the structure and perpendicular to the current flow direction. The variation of the planar Hall resistance was calculated from the Hall voltage, subtracting the offset coming from the longitudinal contribution and normalizing the peaks at ±1. Saturation magnetization values of 750 kA/m and 1120 kA/m were determined for Py and Co, respectively, through magnetization hysteresis loop measurements.

For specific samples, magnetic domains were visualized using X-ray photoemission electron microscopy (XPEEM) at the UE49PGMa/SPEEM beamline at the BESSY II electron storage ring OR (lab) operated by the Helmholtz-Zentrum Berlin für Materialien und Energie. Magnetic contrast was obtained from X-ray magnetic circular dichroism (XMCD) measurements performed at the Co and Fe L_3_-edges.

### Modeling

Micromagnetic simulations were performed using the open-source software Mumax^3^,^43^ a finite difference method to simulate the time- and space-dependent magnetization evolution by solving the Landau–Lifshitz–Gilbert (LLG) equation for the magnetic moments under the effect of an effective field including the exchange, the Zeeman and the dipolar contributions.^44,45^ The material parameters used were the exchange constant, *A*_ex_ = 13 pJ/m, and saturation magnetization *M*_s_ = 860 kA/m, for Py, and *A*_ex_ = 10 pJ/m and *M*_s_ = 1400 kA/m for Co. The discretization cell size is set to 10 nm for each direction. To reduce computation time, the dimensions of the simulated structures have been scaled down compared to the experimental devices, while selecting carefully both the aspect ratio (to limit the difference of demagnetization factor between experiment and simulation) and the sample thickness to have the same kind of domain walls.^46^Table 2 summarizes the dimensions of all the samples used for simulation. Additionally, to prevent unrealistic results induced by a numeric perfect symmetry of the device, a random uniform deviation of a maximum of 2% in the direction of the applied field was considered.

## Supporting Information Available

Magnetic hysteresis loops and thickness dependence of the coercive field of permalloy thin films (Figure S1); analysis of the effect of the petals’ length (Figure S2); analysis of the impact of the number of petals (Figure S3) (PDF)

## References

[ref1] Magnetic Domains; Hubert A.; Schäfer R., Eds.; Springer, 1998.

[ref2] Topology in Magnetism; Zang J.; Cros V.; Hoffmann A., Eds.; Springer Series in Solid-State Sciences, 2018; Vol. 192, pp 1–416.

[ref3] Wang S.; Xu J.; Li W.; Sun S.; Gao S.; Hou Y. Magnetic Nanostructures: Rational Design and Fabrication Strategies toward Diverse Applications. Chem. Rev. 2022, 122, 5411–5475. 10.1021/acs.chemrev.1c00370. 35014799

[ref4] Fernández-Pacheco A.; Streubel R.; Fruchart O.; Hertel R.; Fischer P.; Cowburn R. P. Three-dimensional nanomagnetism. Nat. Commun. 2017, 8, 15756 10.1038/ncomms15756. 28598416 PMC5494189

[ref5] Elahi E.; Khan M. A.; Suleman M.; Dahshan A.; Rehman S.; Khalil H. M. W.; Rehman M. A.; Hassan A. M.; Koyyada G.; Kim J. H.; Khan M. F. Recent innovations in 2D magnetic materials and their potential applications in the modern era. Mater. Today 2024, 72, 183–206. 10.1016/j.mattod.2023.11.008.

[ref6] Peixoto L.; Magalhães R.; Navas D.; Moraes S.; Redondo C.; Morales R.; Araújo J. P.; Sousa C. T. Magnetic nanostructures for emerging biomedical applications. Appl. Phys. Rev. 2020, 7, 011310 10.1063/1.5121702.

[ref7] Matsukura F.; Tokura Y.; Ohno H. Control of magnetism by electric fields. Nat. Nanotechnol. 2015, 10, 209–220. 10.1038/nnano.2015.22. 25740132

[ref8] Lalieu M. L. M.; Lavrijsen R.; Koopmans B. Integrating all-optical switching with spintronics. Nat. Commun. 2019, 10, 110 10.1038/s41467-018-08062-4. 30631067 PMC6328538

[ref9] Bandyopadhyay S.; Atulasimha J.; Barman A. Magnetic straintronics: Manipulating the magnetization of magnetostrictive nanomagnets with strain for energy-efficient applications. Appl. Phys. Rev. 2021, 8, 041323 10.1063/5.0062993.

[ref10] Cowburn R. P. Property variation with shape in magnetic nanoelements. J. Phys. D:Appl. Phys. 2000, 33, R1 10.1088/0022-3727/33/1/201.

[ref11] Lin G.; Makarov D.; Schmidt O. G. Magnetic sensing platform technologies for biomedical applications. Lab Chip 2017, 17, 1884–1912. 10.1039/C7LC00026J. 28485417

[ref12] Zheng C.; et al. Magnetoresistive Sensor Development Roadmap. IEEE Trans. Magn. 2019, 55, 1–30. 10.1109/TMAG.2019.2896036.

[ref13] Tumanski S. Handbook of Magnetic Measurements; Jones B.; Huang H., Eds.; CRC Press, 2011; pp 1–382.

[ref14] Kulkarni P. D.; Iwasaki H.; Nakatani T. The Effect of Geometrical Overlap between Giant Magnetoresistance Sensor and Magnetic Flux Concentrators: A Novel Comb-Shaped Sensor for Improved Sensitivity. Sensors 2022, 22, 9385 10.3390/s22239385. 36502082 PMC9740504

[ref15] Cardoso S.; Leitao D. C.; Gameiro L.; Cardoso F.; Ferreira R.; Paz E.; Freitas P. P. Magnetic tunnel junction sensors with pTesla sensitivity. Microsyst. Technol. 2014, 20, 793–802. 10.1007/s00542-013-2035-1.

[ref16] Zhang X.; Bi Y.; Chen G.; Liu J.; Li J.; Feng K.; Lv C.; Wang W. Influence of size parameters and magnetic field intensity upon the amplification characteristics of magnetic flux concentrators. AIP Adv. 2018, 8, 125222 10.1063/1.5066271.

[ref17] Khan M. A.; Sun J.; Li B.; Przybysz A.; Kosel J. Magnetic sensors-A review and recent technologies. Eng. Res. Express 2021, 3, 022005 10.1088/2631-8695/ac0838.

[ref18] Liu W.; Li Z.; Ansari M. A.; Cheng H.; Tian J.; Chen X.; Chen S. Design Strategies and Applications of Dimensional Optical Field Manipulation Based on Metasurfaces. Adv. Mater. 2023, 35, 2208884 10.1002/adma.202208884. 37055931

[ref19] Ju R.; Xu G.; Xu L.; Qi M.; Wang D.; Cao P.; Xi R.; Shou Y.; Chen H.; Qiu C.; Li Y. Convective Thermal Metamaterials: Exploring High-Efficiency, Directional, and Wave-Like Heat Transfer. Adv. Mater. 2023, 35, 2209123 10.1002/adma.202209123. 36621882

[ref20] Qiu C. W.; Zhang T.; Hu G.; Kivshar Y. Quo Vadis, Metasurfaces?. Nano Lett. 2021, 21, 5461–5474. 10.1021/acs.nanolett.1c00828. 34157842

[ref21] Zheludev N. I.; Kivshar Y. S. From metamaterials to metadevices. Nat. Mater. 2012, 11, 917–924. 10.1038/nmat3431. 23089997

[ref22] Magnus F.; Wood F.; Moore J.; Morrison K.; Perkins G.; Fyson J.; Wiltshire M. C.; Caplin D.; Cohen L. F.; Pendry J. B. A d.c. magnetic metamaterial. Nat. Mater. 2008, 7, 295–297. 10.1038/nmat2126. 18297077

[ref23] Jung P.; Ustibov A.; Anlage S. Progress in superconducting metamaterials. Supercond. Sci. Technol. 2014, 27, 073001 10.1088/0953-2048/27/7/073001.

[ref24] Xu L. J.; Huand J. Magnetostatic chameleonlike metashells with negative permeabilities. EPL (Europhys. Lett.) 2019, 125, 64001 10.1209/0295-5075/125/64001.

[ref25] Navau C.; Prat-Camps J.; Sanchez A. Magnetic energy harvesting and concentration at a distance by transformation optics. Phys. Rev. Lett. 2012, 109, 263903 10.1103/PhysRevLett.109.263903. 23368564

[ref26] Narayana S.; Sato Y. DC magnetic cloak. Adv. Mater. 2012, 24, 71–74. 10.1002/adma.201104012. 22114004

[ref27] Gömöry F.; Solovyov M.; Soucl J.; Navau C.; Prat-Camps J.; Sanchez A. Experimental Realization of a Magnetic Cloak. Science 2012, 335, 1466–1468. 10.1126/science.1218316. 22442477

[ref28] Wang R.; Mei Z.; Cui T. A carpet cloak for static magnetic field. Appl. Phys. Lett. 2013, 102, 213501 10.1063/1.4808013.

[ref29] Zhu J.; Jiang W.; Liu Y.; Yin G.; Yuan J.; He S.; Ma Y. Three-dimensional magnetic cloak working from d.c. to 250 kHz. Nat. Commun. 2015, 6, 8931 10.1038/ncomms9931. 26596641 PMC4696515

[ref30] Prat-Camps J.; Navau C.; Sanchez A. Experimental realization of magnetic energy concentration and transmission at a distance by metamaterials. Appl. Phys. Lett. 2014, 105, 234101 10.1063/1.4903867.

[ref31] Navau C.; Mach-Batlle R.; Parra A.; Prat-Camps J.; Laut S.; Del-Valle N.; Sanchez A. Enhancing the sensitivity of magnetic sensors by 3D metamaterial shells. Sci. Rep. 2017, 7, 44762 10.1038/srep44762. 28303951 PMC5355979

[ref32] Prat-Camps J.; Navau C.; Sanchez A. Quasistatic Metamaterials: Magnetic Coupling Enhancement by Effective Space Cancellation. Adv. Mater. 2016, 28, 4898–4903. 10.1002/adma.201506376. 27120801

[ref33] Fourneau E.; Arregi J. A.; Barrera A.; Nguyen N. D.; Bending S.; Sanchez A.; Uhlíř V.; Palau A.; Silhanek A. V. Microscale Metasurfaces for On-Chip Magnetic Flux Concentration. Adv. Mater. Technol. 2023, 8, 2300177 10.1002/admt.202300177.

[ref34] Lejeune N.; Fourneau E.; Barrera A.; Morris O.; Leonard O.; Arregi J.; Navau C.; Uhlir V.; Bending S.; Palau A.; Silhanek A. Dimensional crossover of microscopic magnetic metasurfaces for magnetic field amplification. APL Mater. 2024, 12, 071126 10.1063/5.0217500.

[ref35] Bort-Soldevila N.; Cunill-Subiranas J.; Barrera A.; Del-Valle N.; Silhanek A. V.; Uhlír V.; Bending S.; Palau A.; Navau C. Enhanced magnetic field concentration using windmill-like ferromagnets. APL Mater. 2024, 12, 021123 10.1063/5.0187035.

[ref36] Barrera A.; Fourneau E.; Martín S.; Batllori J. M.; Alcalá J.; Balcells L.; Mestres N.; Nguyen N. D.; Sanchez A.; Silhanek A. V.; Palau A. Tunable Perpendicular Magnetoresistive Sensor Driven by Shape and Substrate Induced Magnetic Anisotropy. Adv. Sens. Res. 2023, 2, 2200042 10.1002/adsr.202200042.

[ref37] Mor V.; Grosz A.; Klein L. Planar Hall Effect Magnetometers; Grosz A.; Haji-Sheikh M.; Mukhopadhyay S., Eds.; Springer, 2017; Vol. 19.

[ref38] Stoner E. C.; Wohlfarth E. P. Magnetic Recording of Superconducting States. Philos. Trans. R. Soc. A 1948, 204, 599–642. 10.1098/rsta.1948.0007.

[ref39] Bahl C. R. H. Estimating the demagnetization factors for regular permanent magnet pieces. AIP Adv. 2021, 11, 075028 10.1063/5.0060897.

[ref40] Brown W. F. Jr Virtues and weaknesses of the domain concept. Rev. Mod. Phys. 1945, 17, 15 10.1103/RevModPhys.17.15.

[ref41] Voltan S.; Cirillo C.; Snijders H. J.; Lahabi K.; García-Santiago A.; Hernández J. M.; Attanasio C.; Aarts J. Emergence of the stripe-domain phase in patterned permalloy films. Phys. Rev. B 2016, 94, 094406 10.1103/PhysRevB.94.094406.

[ref42] Sandig O.; Herrero-albillos J.; Römer F. M.; Friedenberger N. Journal of Electron Spectroscopy and Imaging magnetic responses of nanomagnets by XPEEM. J. Electron Spectrosc. Relat. Phenom. 2012, 185, 365–370. 10.1016/j.elspec.2012.07.005.

[ref43] Vansteenkiste A.; Leliaert J.; Dvornik M.; Helsen M.; Garcia-Sanchez F.; Van Waeyenberge B. The design and verification of MuMax3. AIP Adv. 2014, 4, 107133 10.1063/1.4899186.

[ref44] Fidler J.; Schrefl T. Micromagnetic modelling-the current state of the art. J. Phys. D:Appl. Phys. 2000, 33, R135 10.1088/0022-3727/33/15/201.

[ref45] Brown W. F. Micromagnetics; Interscience Tracts on Physics and Astronomy; Interscience Publishers, 1963.

[ref46] Middelhoek S. Domain walls in thin Ni-Fe films. J. Appl. Phys. 1963, 34, 1054–1059. 10.1063/1.1729367.

