# Supplementary material for: On-Chip Planar Metasurfaces for Magnetic Sensors with Greatly Enhanced Sensitivity
Source: ACS Nano. 2025 Mar 6;19(10):10461–75. doi: 10.1021/acsnano.5c00422 (PMC11924339; doi:10.1021/acsnano.5c00422)
Supplement: Supplementary file 1 — nn5c00422_si_001.pdf [file nn5c00422_si_001.pdf]

On-Chip Planar Metasurfaces for Magnetic Sensors with Greatly Enhanced Sensitivity

Aleix Barrera,[†,@] Emile Fourneau,[*,‡,@] Natanael Bort-Soldevila,[¶] Jaume Cunill-Subiranas,[¶] Nuria Del-Valle,[¶] Nicolas Lejeune,[‡] Michal Staňo,[§] Alevtina Smekhova,[‖] Narcis Mestres,[†] Lluis Balcells,[†] Carles Navau,[¶] Vojtěch Uhlíř,[§,⊥] Simon J. Bending,[#] Sergio Valencia,[‖] Alejandro V. Silhanek,[*,‡] and Anna Palau[*,†]

†*Institut de Ciència de Materials de Barcelona, ICMAB-CSIC, Spain.*

‡*Experimental Physics of Nanostructured Materials, Department of Physics, Universitè de Liège, B-4000 Sart Tilman, Belgium.*

¶*Departament de Física, Universitat Autònoma de Barcelona, 08193 Bellaterra, Spain.*

§*CEITEC BUT, Brno University of Technology, Purkyňova 123, Czech Republic.*

‖*Helmholtz-Zentrum Berlin fur Materialien und Energie, Albert-Einstein-Strasse 15, D-12489 Berlin, Germany.*

⊥*Institute of Physical Engineering, Brno University of Technology, Technická 2, 616 69 Brno, Czech Republic.*

#*Centre for Nanoscience and Nanotechnology, Department of Physics, University of Bath, Bath, BA2 7AY, United Kingdom.*

@*Equally contributed to this work*

E-mail: Emile.Fourneau@uliege.be; asilhanek@uliege.be; palau@icmab.es

Thickness dependence of the coercive field of permalloy thin films

Evolution of the coercive field, H_c, of permalloy thin films with different thicknesses. An abrupt change of H_c is observed for films with thickness larger than 120 nm due to the formation of stripe-like magnetic domains.

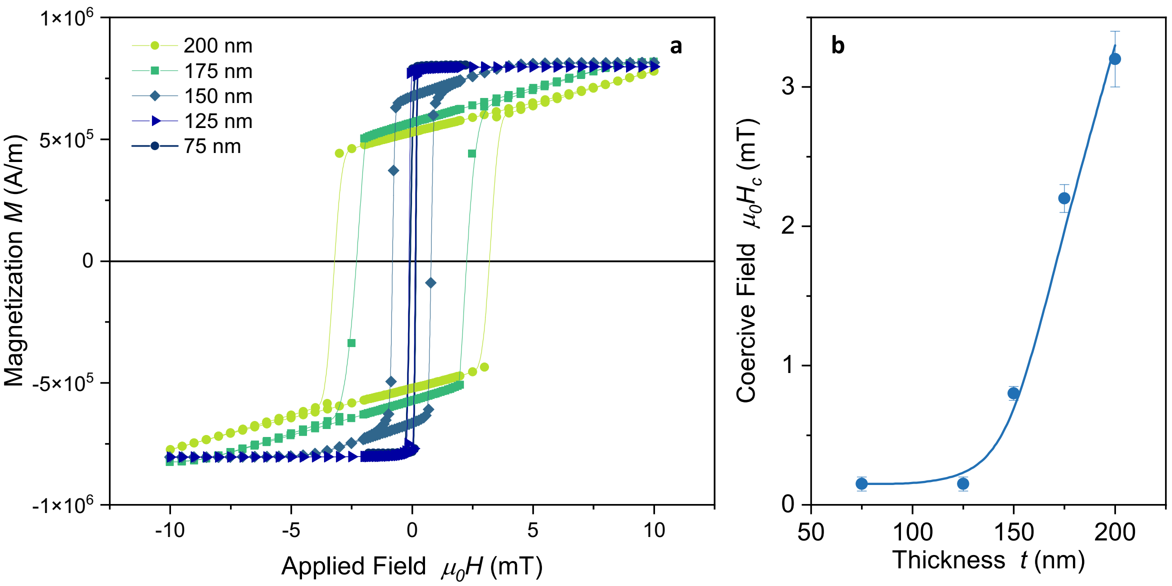

Figure S1. Magnetization of Permalloy thin films of different thicknesses. (a) Magnetic hysteresis loops obtained for Permalloy thin films of different thicknesses. (b) Thickness dependence of the coercive field.

Effect of the petals' length

Increasing the length of the petals has two significant effects. Firstly, longer petals result in a higher concentration effect due to the increased amount of magnetic material. Secondly, the pinning effect of the petals on the magnetic domains within the core is greatly influenced by the length of the petals. As illustrated in Fig. S2, shorter petals lead to domain arrangements forming a Landau pattern, whereas the larger aspect ratio of longer petals pins the domains at the apex along their easy axis. Consequently, both the coercive field of the structure and the susceptibility induced by the abrupt switching of the petals increase with the length of the petals.

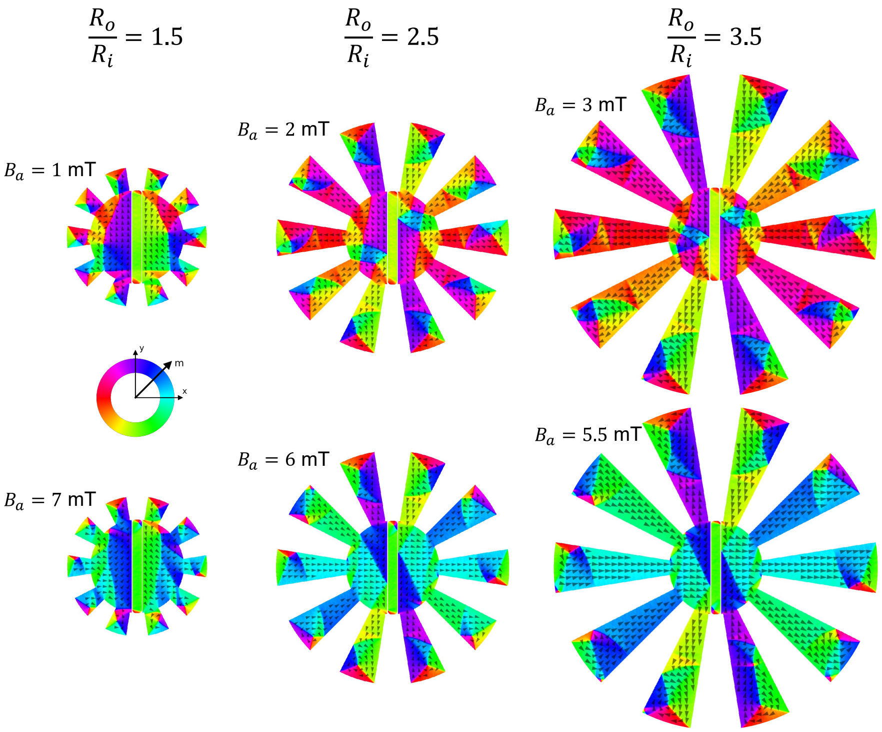

Figure S2. Micromagnetic simulations showing the effect of the radii ratio. Top panels show the magnetic domains arrangement at different increasing applied magnetic field in a device with a small radii ratio $R_\mathrm{o}/R_\mathrm{i} = 1.5$, while the bottom panels correspond to a larger radii ratio $R_\mathrm{o}/R_\mathrm{i} = 3.5$.

Effect of the number of petals

The effective susceptibility of the MFCs exhibits a bell-shaped response with respect to the number of petals, N_p (See Fig.4(f) and Fig.6(c) of the main manuscript). As shown in Fig. S3, this phenomenon is associated with the decrease in the mean width of the petals as their number increases (We adopt here the rule of equal size for the gaps between consecutive petals than for the petals). With wider petals ($N_p = 2$), the aspect ratio is reduced, resulting in magnetic domains relaxing into flux-closed patterns, leading to a weaker susceptibility. Conversely, narrower petals ($N_p = 6$) exhibit abrupt switching, offering a higher susceptibility. However, very narrow petals ($N_p = 26$) exert less influence on the central core of the device. Consequently, since the switching field of each petal depends on the orientation of their easy axis relative to the applied field direction and the presence of any potential defects (random distribution of saturation magnetization in the micromagnetic simulation), the magnetic reversal of the device occurs over a broader range of external magnetic fields, leading to a reduced magnetic susceptibility.

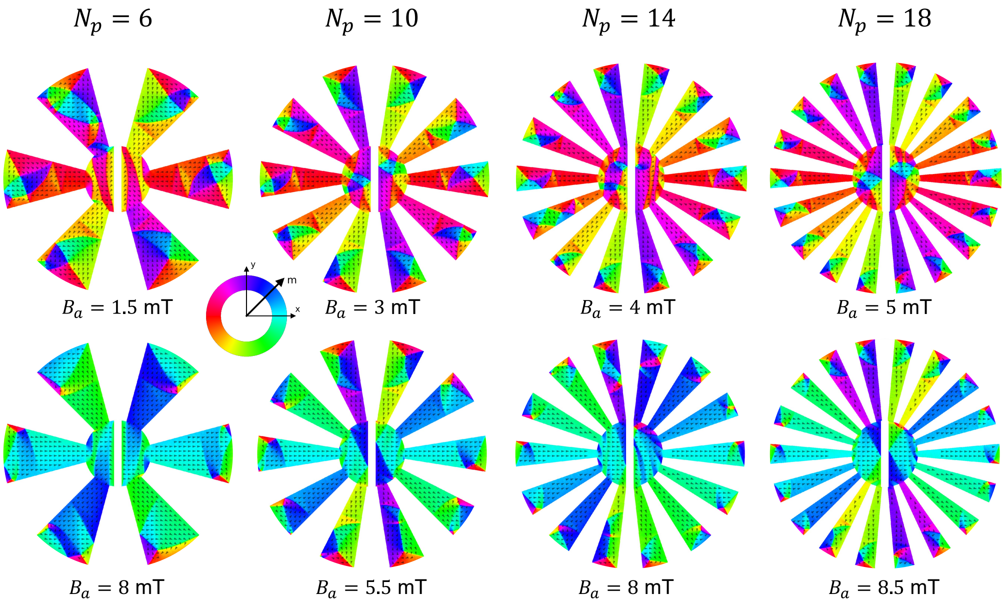

Figure S3. Micromagnetic simulations showing the effect of the number of petals. Panels show the magnetic domains arrangement at different increasing applied magnetic field (from left to right) in devices with 2, 6 and 26 petals (from top to bottom).
